# Enhancing Conversion Kinetics through Electron Density Dual‐Regulation of Catalysts and Sulfur toward Room‐/Subzero‐Temperature Na–S Batteries

**DOI:** 10.1002/advs.202308180

**Published:** 2024-04-09

**Authors:** Sainan Luo, Jiafeng Ruan, Yan Wang, Min Chen, Limin Wu

**Affiliations:** ^1^ Department of Materials Science Fudan University Shanghai 200433 P. R. China; ^2^ School of Materials and Chemistry University of Shanghai for Science and Technology Shanghai 200093 P. R. China

**Keywords:** dual‐modulating strategy, electron density, selenium, sodium‐sulfur batteries, ZnS

## Abstract

Room‐temperature sodium–sulfur (RT Na/S) batteries have received increasing attention for the next generation of large‐scale energy storage, yet they are hindered by the severe dissolution of polysulfides, sluggish redox kinetic, and incomplete conversion of sodium polysulfides (NaPSs). Herein, the study proposes a dual‐modulating strategy of the electronic structure of electrocatalyst and sulfur to accelerate the conversion of NaPSs. The selenium‐modulated ZnS nanocrystals with electron rearrangement in hierarchical structured spherical carbon (Se‐ZnS/HSC) facilitate Na^+^ transport and catalyze the conversion between short‐chain sulfur and Na_2_S. And the in situ introduced Se within S can enhance conductivity and form an S─Se bond, suppressing the “polysulfides shuttle”. Accordingly, the S@Se‐ZnS/HSC cathode exhibits a specific capacity of as high as 1302.5 mAh g^−1^ at 0.1 A g^−1^ and ultrahigh‐rate capability (676.9 mAh g^−1^ at 5.0 A g^−1^). Even at −10 °C, this cathode still delivers a high reversible capacity of 401.2 mAh g^−1^ at 0.05 A g^−1^ and 94% of the original capacitance after 50 cycles. This work provides a novel design idea for high‐performance Na/S batteries.

## Graphical abstract

The selenium‐modulated ZnS nanocrystals with electron rearrangement in hierarchical structured spherical carbon (Se‐ZnS/HSC) facilitate Na^+^ transport and catalyze conversion between short‐chain sulfur and Na_2_S. As expected, S@Se‐ZnS/HSC cathode exhibits ultrahigh‐rate capability (676.9 mAh g^−1^ at 5.0 A g^−1^) and high reversible capacity of 401.2 mAh g^‐1^ at 0.05 A g^−1^ even at −10 °C.

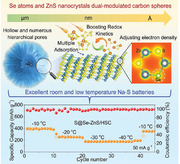

## 1 Introduction

Recently, the room‐temperature sodium‐sulfur (RT Na/S) battery has attracted enormous attention on account of its high energy density (1274 Wh kg^−1^), high specific capacity (1675 mAh g^−1^) of sulfur, and abundant resources of sodium and sulfur.^[^ ^1^, ^2^, ^3^ ^]^ However, this battery system faces several major challenges, which severely impede its further development. First, the insulating nature of sulfur and the discharge end product of Na_2_S result in sluggish reaction kinetics and large polarization.^[^ ^4^ ^]^ Second, severe volumetric expansion (170%) during sodiation leads to the exfoliation and inactivation of actives, causing fast capacity degradation.^[^ ^5^ ^]^ Third, the dissolution of sodium polysulfides (NaPSs) in liquid electrolytes produces a serious “shuttle effect” and self‐discharge.^[^ ^6^ ^]^ These issues cause not only low sulfur utilization but also fast capacity fade and inferior rate capability of RT Na/S batteries.^[^ ^7^ ^]^

To work out these issues, some rational strategies have been employed to promote the conversion and anchoring of NaPSs. Especially, the carbon matrix has been proven to facilitate electron transport and physically confine NaPSs.^[^ ^2^ ^]^ However, it must be pointed out that non‐polar carbon matrices do not effectively anchor soluble NaPSs due to weak van der Waals forces, which results in gradual capacity decay.^[^ ^8^, ^9^ ^]^ Besides, the lack of catalytic sites in the carbon host makes it unable to promote the complete reduction of NaPSs, which still forms a low reversible capacity. Recently, polar transition metal sulfides (TMS) with intrinsic affinity to NaPSs are introduced into carbon matrices to alleviate the “shuttle effect” and accelerate the conversion kinetics of NaPSs based on the strong polarity and catalytic activity.^[^ ^10^, ^11^ ^]^ For example, Yan et al. reported on the fabrication of N‐doped porous carbon nanotubes with NiS_2_ nanocrystals (NiS_2_@NPCTs),^[^ ^12^ ^]^ wherein the closed porous structure had a physical restriction on polysulfides, while the polarized NiS_2_ promotes the conversion and immobilization of NaPSs. The obtained NiS_2_@NPCTs/S cathode could display a reversible capacity of 650 mAh g^−1^ at 0.1 A g^−1^ after 200 cycles. Aslam et al. designed the hollow polar catalytic CoS_2_/C as an efficient host of sulfur,^[^ ^13^ ^]^ withstanding volume expansion and effectively accelerating the conversion of NaPSs. Nevertheless, the rate performance and utilization of sulfur are still far from satisfactory due to the unsatisfied catalytic activity and poor conductivity of these introduced TMS catalysts. The catalytic activity and intrinsic conductance of TMS are closely related to their electronic structure.

Besides, another nearly neglected rate‐limiting factor of RT Na/S battery is the sluggish reaction kinetic in the conversion of NaPSs due to the insulating nature of sulfur (5 × 10^−28^ S m^−1^) and Na_2_S.^[^ ^14^ ^]^ Therefore, it is important to optimize the intrinsic activity of sulfur for boosting reaction kinetics. Similar to sulfur, selenium can be alloyed with sodium for sodium ion (Na^+^) storage. More importantly, it is expected to exhibit faster kinetics than sulfur due to the higher electronic conductivity of selenium (1 × 10^−3^ S m^−1^).^[^ ^15^ ^]^ Although selenium shows a lower theoretical specific capacity (675 mAh g^−1^),^[^ ^16^, ^17^ ^]^ introducing a small amount of selenium into sulfur can effectively enhance its electronic conductivity without significantly reducing its capacity.^[^ ^18^ ^]^ Besides, the chemical bonding formed between selenium and sulfur addresses the dissolution issue of NaPSs.^[^ ^19^, ^20^ ^]^ However, most studies focus on either the electronic structure of polar catalysts or the intrinsic activity of sulfur, ignoring the synergistic mechanism. Actually, both the reaction energy barrier and conductivity greatly affect the polysulfide conversion, which could be modulated by adjusting the electronic structure of polar catalysts and sulfur, respectively. Therefore, the rational design of sulfur cathode focusing on the electronic structure of catalysts and intrinsic activity of sulfur simultaneously for RT Na/S batteries is promising but remains challenging.

In this work, we propose a novel dual‐modulating strategy by adjusting the electron density of sulfur and zinc sulfide (ZnS) catalysts simultaneously using an in situ displacement reaction. The modified sulfur and selenium‐zinc sulfide (Se‐ZnS) nanocrystals confined in hollow and hierarchical carbon spheres (S@Se‐ZnS/HSC) are prepared by annealing treatment and sulfur vapor infiltration. Benefiting from the introduction of selenium, the Se‐ZnS nanocrystals with electron rearrangement show strong chemical bonding and improved redox properties of NaPSs, causing high utilization of sulfur. Meanwhile, selenium is introduced in sulfur to modulate the electronic structure of sulfur pieces, thus improving the conductivity and intrinsic activity of NaPSs. Additionally, these interconnected carbon skeletons with numerous hierarchical pores not only ensure 68 wt.% of high sulfur loading, but also adapt to volume changes, and promote the accessibility of the Se‐ZnS catalysts to polysulfides. Accordingly, the S@Se‐ZnS/HSC cathodes display a high specific capacity of 1302.5 mAh g^−1^ at 0.1 A g^−1^ and an excellent rate capability of 676.9 mAh g^−1^ at 5.0 A g^−1^, which are obviously superior to those of the previously reported cathode materials, showing very promising prospects for practical RT Na/S batteries.

## 2 Results and Discussion

### 2.1 Preparation and Characterization of Sulfur Cathodes

**Figure 1:**
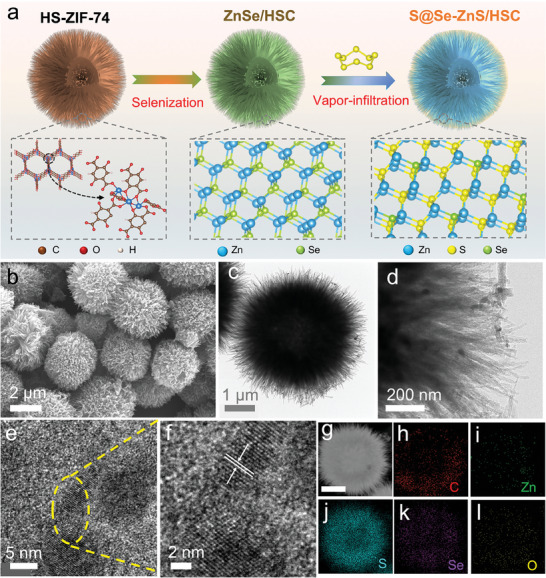
Preparation and morphology of S@Se‐ZnS/HSC: a) the preparation process of the S@Se‐ZnS/HSC. b) SEM image of S@Se‐ZnS/HSC. c,d) TEM image. e,f) HRTEM image. g) High‐angle annular dark‐field STEM image of the S@Se‐ZnS/HSC. h,i) EDS elemental mapping of C, Zn, S, Se, and O.

The fabrication process of S@Se‐ZnS/HSC composites was briefly shown in **Figure** **1**a and Figure S1 (Supporting Information). First, hierarchical spherical zinc‐MOF‐74 (HS‐ZIF‐74) precursors were synthesized by coprecipitation and hydrothermal process.^[^ ^21^ ^]^ Specifically, 1D ZIF‐74 nanocrystals were generated from zinc acetate dihydrate and 2,5 dihydroxy terephthalic acid in a methanol solution,^[^ ^22^ ^]^ and were subsequently assembled into 3D hierarchical and spherical structure samples by using urea as the modifier. Second, the obtained HS‐ZIF‐74 precursors were converted into ZnSe‐ZnO/HSC after high‐temperature selenization. The hierarchical porous spherical carbon with ZnSe nanocrystals (ZnSe/HSC) was obtained after the removal of ZnO with hydrogen fluoride etching. Finally, sulfur was loaded into the pores and the cavities of the ZnSe/HSC host by vapor diffusion. Meanwhile, the selenium in ZnSe/HSC is gradually replaced by sulfur in a quartz tube, forming the objective product (S@Se‐ZnS/HSC). The S@ZnS/HSC and S@HSC were fabricated by sulfur infiltration using the ZnS/HSC and HSC as hosts for comparison, respectively.

The HS‐ZIF‐74 precursor spheres (≈5–7 µm) were composed of 1D ZIF nanorods, which are uniformly distributed, as shown in Figure S2 (Supporting Information). After carbonization and sulfur loading, the obtained S@Se‐ZnS/HSC still maintains a hierarchical spherical structure (Figure 1b,c). The S@ZnS/HSC and S@HSC showed diameters similar to that of the HS‐ZIF‐74 precursor spheres (Figure S3, Supporting Information). In general, micron‐sized S@Se‐ZnS/HSC obtained higher volumetric energy density than nano‐sized materials.^[^ ^23^ ^]^ The detailed nanostructures of composites were revealed by high‐resolution TEM (HRTEM). As shown in Figure 1c, S@Se‐ZnS/HSC composites exhibit chestnut‐shell‐like structures with an interior cavity (≈2 µm), which is beneficial to ions transport and sulfur load. The HSC, ZnS/HSC, and ZnSe/HSC showed similar chestnut‐shell‐like structures in Figures S4–S6 (Supporting Information). S@Se‐ZnS/HSC composites show clear surfaces without obvious sulfur particles, indicating successful penetration of sulfur. According to Figure 1d, one can see that the Se‐ZnS nanocrystals with an average size of ≈15 nm (Figure 1e,f) are uniformly dispersed in these 1D porous carbon nanorods, which can improve the utilization of Se‐ZnS catalysts. The measured lattice spacing is 0.31 nm, which is well attributed to the (111) crystal plane of ZnS.^[^ ^24^ ^]^ Energy‐dispersive X‐ray spectroscopy (EDS) mapping of S@Se‐ZnS/HSC in Figure 1g–l reveals the uniform dispersion of sulfur and Se‐ZnS nanocrystals in the carbon spheres.

**Figure 2:**
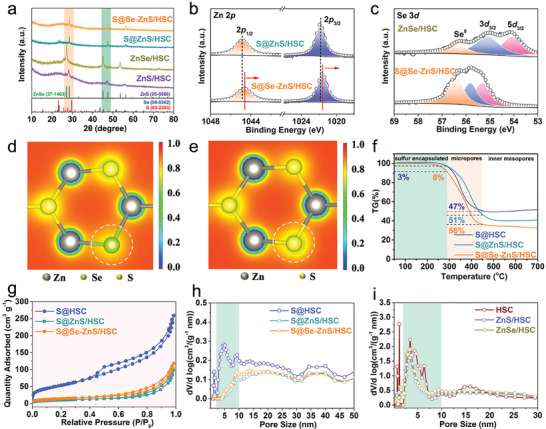
Structure characterization of hosts and sulfur composites: a) XRD patterns of the ZnS/HSC, ZnSe/HSC, S@ZnS/HSC, and S@Se‐ZnS/HSC. b) XPS survey spectrum of ZnS/HSC, ZnSe/HSC, S@ZnS/HSC, and S@Se‐ZnS/HSC. c) High‐resolution XPS spectrum of Se 3*d*. d) Electronic structure differences for Se‐ZnS. e) Electronic structure differences for ZnS. f) TGA curves. g) N_2_ adsorption/desorption isotherms. h,i) Pore size distribution.

The X‐ray powder diffraction of ZnSe/HSC (XRD, **Figure** **2**a) indicates the diffraction peaks at 27.2°, 45.2°, and 53.6° are indexed to (111), (220), and (311) crystal planes of ZnSe (PDF# 37–1463).^[^ ^25^ ^]^ For ZnS/HSC, S@ZnS/HSC, and S@Se‐ZnS/HSC composites, there were also three characteristic peaks at 28.5°, 47.5°, and 56.2°, corresponding to (111), (220), and (311) crystal planes of the ZnS (PDF# 05–0566), respectively.^[^ ^26^ ^]^ Besides, selenium doping might be inducing amorphous regions or creating defects in the lattice, which may not be evident in the crystalline structure observed by XRD or HRTEM. In Figure 2a, there is small peak ≈23° for S@ZnS/HSC, which can be indexed to the (222) crystal planes of S_8_ (PDF# 83–2283). No obvious sulfur characteristic peaks in S@Se‐ZnS/HSC and S@HSC, indicate that sulfur has been penetrated into the interior cavity and micro/mesopores (Figure 2a; Figure S7, Supporting Information).^[^ ^27^ ^]^ The as‐prepared composite also displayed two characteristic peaks related to the D (≈1345 cm^−1^) and G‐band(1576 cm^−1^) signals of carbon (Figure S8, Supporting Information), respectively.^[^ ^28^ ^]^ No characteristic peaks of ZnS or ZnSe suggest that they have been dispersed in the carbon host with very small sizes.^[^ ^29^ ^]^ Besides, no signal of sulfur is observed, confirming that sulfur has been infiltrated into the interior of hosts. The Zn 2*p* XPS scan curves (Figure 2b,c; Figure S9, Supporting Information) of S@ZnS/HSC show one doublet located at 1021.4 eV (Zn 2*p*3/2) and 1044.1 (Zn 2*p*1/2), which is assigned to the chemical environment of Zn^2+^ within ZnS. All of the binding energies of these Zn 2*p* peaks in S@Se‐ZnS/HSC are slightly lower than those of S@ZnS/HSC, demonstrating that the electron density around the Zn sites in S@Se‐ZnS/HSC is increased. This slight shift might be due to the residual Se in ZnS after replacement between ZnSe and sulfur during the sulfur loading.^[^ ^30^ ^]^ Excess electrons are redistributed between adjacent atoms after the introduction of Se in ZnS, which correspondingly increases the electrical conductivity. Two peaks in the S 2*p* spectrum 164.0 and 162.9 eV are ascribed to the S 2*p*1/2 and S 2*p*3/2 in ZnS. Furthermore, two peaks at ≈164.7 and 163.4 eV, correspond to the S 2*p*1/2 and S 2*p*3/2 in elemental sulfur. A minor peak corresponding to C−SO_x_ groups is observed at 168.7 eV.^[^ ^31^ ^]^ The Se 3*d* spectrum of ZnSe/HSC displays three peaks at 54.5, 55.4, and 55.9 eV assigned to the Se 3*d*5/2, Se 3*d*3/2, and Se^0^, respectively (Figure 2c).^[^ ^32^ ^]^ Note that the XPS Se 3*d* peak of S@Se‐ZnS/HSC shifts to higher energy. The reason for this change might be attributed to the electronegativity difference between sulfur and selenium.^[^ ^33^ ^]^ The electronic structure differences between ZnS and Se‐ZnS can be visually mapped in Figure 2d,e. After selenium atoms enter the lattice of ZnS, fewer electrons in the Zn atoms tend to flow toward the selenium atom, decreasing the negative charge of selenium and increasing the electron density of the metal. The Zn atoms that have excess electrons are considered as the centers of negative charge to attract NaPSs.^[^ ^34^ ^]^ To investigate the elemental ratios of selenium and sulfur in the S@Se‐ZnS/HSC, inductively coupled plasma‐optical emission spectrometry (ICP‐OES) was used to analysis the samples. The results indicated that the elemental ratios of sulfur and selenium in S@Se‐ZnS/HSC were measured at 10:1. Since part of the sulfur combined with zinc to form zinc sulfide, the residual elemental ratio of sulfur to selenium was ≈9:1.

The sulfur contents in composites were measured by thermogravimetric analysis (TGA). As shown in Figure 2f, the sulfur contents of the S@HSC, S@ZnS/HSC, and S@Se‐ZnS/HSC samples are calculated to be 50.1, 60.4, and 68.2 wt.%, respectively. Most of the sulfur (≈56 wt.%) in S@Se‐ZnS/HSC composites evaporated at 290 to 450 °C, which means it is confined in the micropores with amorphous nature.^[^ ^35^ ^]^ The highest loading of amorphous sulfur in S@Se‐ZnS/HSC suggests that the Se‐ZnS nanocrystals with polar surfaces can assist the carbon host to capture sulfur, thus increasing sulfur loading. The ZnSe/HSC, ZnS/HSC, and HSC display large specific surface areas (1115.1, 1125.8, and 1515.8 m^2^ g^−1^, respectively) and hierarchical porous structure (Figure 2g,h), which can provide sufficient space for loading sulfur and alleviating volume expansion.^[^ ^36^ ^]^ After infiltrating sulfur into hosts, the specific surface areas of the S@Se‐ZnS/HSC, S@ZnS/HSC, and S@HSC are decreased to 48.8, 34.8, and 186.2 m^2^ g^−1^, respectively (Figure 2i; Figure S10, Supporting Information). These results indicate the successful permeation of sulfur into the interior pores and cavities of hosts. Although the numbers of micro/mesopores are significantly reduced, some mesopores of S@Se‐ZnS/HSC still remain, which could promote the penetration of electrolytes and alleviate volume expansion.^[^ ^37^ ^]^

### 2.2 Electrochemical Properties of the S@Se‐ZnS/HSC Cathodes

**Figure 3:**
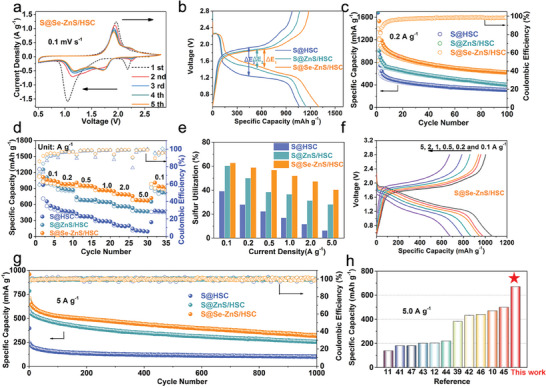
Electrochemical performance: a) CV of the S@Se‐ZnS/HSC at the initial five cycles. b) Galvanostatic discharge profiles of the S@HSC, S@ZnS/HSC, and S@Se‐ZnS/HSC cathode at 0.1 A g^−1^. c) Cycling performance at 0.2 A g^−1^. d) Rate performance. e) The sulfur utilization at different current densities. f) Discharge/charge curves at different current densities. g) Cycling performance at 5.0 A g^−1^. h) Rate comparison of this work and reported cathode materials for RT Na/S batteries.

The electrochemical properties of the S@Se‐ZnS/HSC cathode were evaluated in coin cells with sodium foils as anodes. **Figure** **3**a displays the typical cyclic voltammetry (CV) curves of the S@Se‐ZnS/HSC electrode in the voltage window of 0.5–2.8 V at 0.1 mV s^−1^. Two reduction peaks centered at 2.15 and 1.05 V are observed in the first cathodic scan. These cathode peaks correspond to the reduction from elemental sulfur to long‐chain NaPSs and the subsequent formation of Na_2_S_2_/Na_2_S. In subsequent cycles, a high‐voltage‐plateau region at ≈2.25 V, corresponding to a solid–liquid transition from elemental sulfur to dissolved Na_2_S_8_. And the cathode peaks of S@Se‐ZnS/HSC move to 1.72 and 1.15 V, corresponding to the reversible conversion of long‐chain sodium polysulfides (Na_2_S_x_, x ≥4) to short‐chain polysulfides (Na_2_S_x_, x≤3) and the eventual formation of Na_2_S, respectively.^[^ ^38^ ^]^ For the anodic process, the reversible peak that appeared at 1.89 V and small peak around at 2.25 V were assigned to the reversed transformation from Na_2_S_2_/Na_2_S to long chain NaPSs and sulfur, indicating the excellent electrochemical reversible and deep conversion.^[^ ^39^ ^]^ Besides, the CV curves almost overlap after the 2nd cycle, indicating the good reversibility of the S@Se‐ZnS/HSC electrode. Furthermore, oxidation peaks of the S@Se‐ZnS/HSC exhibit a negative shift while the reduction peaks show a positive shift, suggesting expedited sulfur redox kinetics derived from electron structure dual‐modulation both in sulfur and Se‐ZnS catalyst (Figure S11, Supporting Information). Accordingly, those S@Se‐ZnS/HSC electrodes deliver the lowest voltage gap (∆E) and the highest discharge capacity of 1302.5 mAh g^−1^ at 0.1 A g^−1^ (Figure 3b).^[^ ^40^ ^]^ Figure S12 (Supporting Information) showed the first discharge curves of the S@Se‐ZnS/HSC at 0.1 A g^−1^. The S@Se‐ZnS/HSC exhibited two discharging plateaus ranging from 2.18 to 2.25 V and 1.72 to 1.15 V. These results confirm that S@Se‐Zn S/HSC had the fastest conversion kinetics of NaPSs due to high catalytic activity and improved electrical conductivity. The cycling performances of these electrodes are initially evaluated at 0.2 A g^−1^ as shown in Figure 3c and Figure S13 (Supporting Information). The cycling performance of S@Se‐ZnS/HSC and S@ZnS/HSC, including the activation process at a current density of 0.1 A g^−1^ in the first three cycles. The S@Se‐ZnS/HSC maintains a reversible capacity of 729 mAh g^−1^ and a capacity retention rate of 76% over 100 cycles, higher than that of S@ZnS/HSC and S@HSC. These results demonstrate that more sulfur has been utilized and the shuttle of NaPSs is limited in S@Se‐ZnS/HSC. In addition, cycling performance of the Se‐ZnS/HSC host was conducted. The Se‐ZnS/HSC electrode only exhibits a low specific capacity of ≈22 mAh g^−1^ at 0.2 A g^−1^ within the same potential window (Figure S14, Supporting Information). The result indicated that Se‐ZnS/HSC, serving as a conductive matrix with a catalyst, made a negligible contribution to the total capacity. Figure 3d exhibits the rate performance of the S@Se‐ZnS/HSC, S@ZnS/HSC, and S@HSC composites. A significant reversible capacity of 1064.5, 979.6, 950.1, 869.2, and 792.1 mAh g^−1^ for S@Se‐ZnS/HSC has been reached at 0.1, 0.2, 0.5, 1.0, and 2.0 A g^−1^, respectively. Especially a high capacity of 676.9 mAh g^−1^ with a high sulfur utilization of 40.5% (based on S: 1675 mAh g^−1^) is achieved for the S@Se‐ZnS/HSC at an ultrahigh current density of 5.0 A g^−1^, higher than those of S@ZnS/HSC (477.5 mAh g^−1^, 28.1%) and S@HSC (105.2 mAh g^−1^, 6.3%) in Figure 3e. When the current density switches back to 0.1 A g^−1^, S@Se‐ZnS/HSC can still deliver a high specific capacity of 1007.6 mAh g^−1^. Figure 3f reveals the charge‐discharge curves of the S@Se‐ZnS/HSC. Compared with S@ZnS/HSC and S@HSC, S@Se‐ZnS/HSC electrodes show lower polarization potentials (Figure S15, Supporting Information). It is noteworthy that the S@Se‐ZnS/HSC was able to maintain two well‐maintained plateaus even at 5.0 A g^−1^, implying the high stability and fast reaction kinetics of this electrode. Moreover, the long‐term cycling stability was evaluated under the high current density of 5.0 A g^−1^. The high reversible capacity of 335.5 mAh g^−1^ is obtained even after 1000 cycles. In contrast, the ZnS/HSC and S@HSC cathodes show a lower capacity of 284.6 and 105.9 mAh g^−1^ under the same conditions, respectively (Figure 3g). As shown in Figure S16 (Supporting Information), the structural integrity of the carbon skeleton remains intact without significant changes after cycling. These results further testify that even at high current rates, Se‐ZnS with high catalytic activity and sulfur with improved conductivity can accelerate the reaction kinetics of sulfur species while alleviating the shuttling of NaPSs. The outstanding rate performance of the S@Se‐ZnS/HSC is obviously superior to those of the previously reported cathode materials of RT Na–S batteries (Figure 3h; Figure S17 and Table S1, Supporting Information).^[^ ^10^, ^11^, ^12^, ^39^, ^41^, ^42^, ^43^, ^44^, ^45^, ^46^, ^47^ ^]^

### 2.3 Electrochemical Kinetics and Catalytic Mechanism Characterization

**Figure 4:**
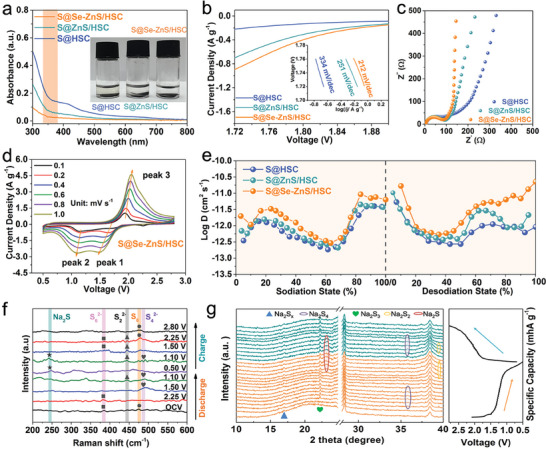
Characterization of electrochemical kinetics and catalytic mechanism: a) the photographs n and its corresponding visible adsorbability of three matrixes to polysulfides. b) Potentiostatic polarization curves and the inset showing the derived Tafel plots. c) Nyquist plots at open‐circuit voltage. d) CV curves of S@Se‐ZnS/HSC electrodes at different scan rates. e) The calculated Na^+^ diffusion coefficient of S@HSC, S@ZnS/HSC and S@Se‐ZnS/HSC cathodes. f) Ex situ Raman analysis of the S@Se‐ZnS/HSC at different discharged voltages. g) In situ XRD patterns of the S@Se‐ZnS/HSC cathode measured at the first cycle.

Visualized tests were taken to detect the adsorption properties of Se‐ZnS/HSC with the NaPSs. The coin cells of S@Se‐ZnS/HSC, S@ZnS/HSC, and S@HSC at different discharge potentials (1.5, 1.1, and 0.5 V) were disassembled, and electrodes were taken out and immersed in electrolyte for 12 h (Figure S18, Supporting Information). The solution containing S@Se‐ZnS/HSC electrodes (sulfur mass loading: 0.99 mg, discharged at 1.1 V during the first cycle) and S@ZnS/HSC (0.98 mg) was almost colorless, while the light‐yellow color was observed in the solution of S@HSC electrodes (0.96 mg, **Figure** **4**a). Additionally, the adsorption properties of samples discharged at 1.1 V could also be confirmed by UV–vis absorption. The weakest signal of polysulfide is detected in the solution containing S@Se‐ZnS/HSC, further proving Se‐ZnS with electron rearrangement possess a strong trapping capability for long‐chain NaPSs. This significant chemisorption can effectively mitigate shuttle effect, which is important for the stability of S@Se‐ZnS/HSC during the long cycling.^[^ ^2^ ^]^ To further reveal the fact that modification of selenium on the catalytic activity of ZnS for the electrochemical conversion kinetic, the linear sweep voltammetry (LSV) tests for sulfur cathodes were conducted. As shown in Figure S19 (Supporting Information) and Figure 4b, two characteristic reduction peaks of sulfur cathodes are easily observed in the LSV curves. The peak centered ant 1.5 V was attributed to the conversion from liquid Na_2_S_x_ (x ≥4) to short‐chain polysulfides (Na_2_S_x_, x≤3), while the peak at 1.1 V corresponded to the formation of insoluble Na_2_S_2_/Na_2_S. The substantially positive shift of peak and lower Tafel slope of S@Se‐ZnS/HSC confirm the effectiveness of the Se‐ZnS catalyst, suggesting better interfacial kinetics of Se‐ZnS/HSC in Na/S batteries.^[^ ^48^ ^]^

To explore the Na^+^ storage mechanism and the reaction kinetics, electrochemical impedance spectroscopy (EIS) measurements, CV at different sweep rates, and galvanostatic intermittent titration technique (GITT) were characterized. Compared to the ZnS/HSC and S@HSC electrodes, the Nyquist spectrum of the S@Se‐ZnS/HSC electrode shows the lowest charge transfer resistance (Figure 4c), suggesting a faster charge transfer ability during the charge/discharge process. These results are attributed to the introduction of high‐conductive elemental selenium and enhancement of the intrinsic electronic conductivity of the Se‐ZnS and sulfur. The CV curves of sulfur cathodes at different scan rates from 0.1 to 1.0 mV s^−1^, as shown in Figure 4d and Figure S20 (Supporting Information), indicate that the b‐value of peak 1, peak 2, and peak 3 are 0.83, 0.83, and 0.78 (Figure S21, Supporting Information). The b‐values of the S@Se‐ZnS as /HSC cathode are closer to 1, higher than those of the S@ZnS/HSC and S@HSC electrodes, indicating more surface reaction and faster reaction speed. The GITT was carried out to further understand the kinetics of the Na^+^ diffusion coefficient. Figure S22 (Supporting Information) displayed the voltage response of the three electrodes during the charge/discharge process. Compared with S@ZnS/HSC and S@HSC cathodes, the S@Se‐ZnS/HSC cathode shows lower overpotential.

The Na^+^ diffusion coefficient (D_Na_^+^) was calculated according to Fick's second law, as detailed in Figure S23 (Supporting Information). As shown in Figure 4e, in the whole voltage range, the D_Na+_ value of S@Se‐ZnS/HSC is higher than those of S@ZnS/HSC and S@HSC, suggesting the enhanced electrochemical kinetics of the S@Se‐ZnS/HSC.^[^ ^49^ ^]^ The improved kinetics performance of S@Se‐ZnS/HSC might be ascribed to the faster conversion of NaPSs in modified Se‐ZnS/HSC hosts, supporting a superior rate capability. To detect the polysulfide conversion in the discharge process, ex situ Raman of the S@Se‐ZnS/HSC was further explored at different voltages (original, 2.25, 1.7, 1.5, 1.1, and 0.5 V, Figure 4f). The S_8_ peaks can be detected before the discharge process. During the discharge process, peaks corresponding to S^x−^ (x = 2–6) can be observed during the charge process from open circuit voltage (OCV) to 1.1 V. When the cell was discharged to ≈0.5 V, the signal of Na_2_S can be detected.^[^ ^12^, ^35^ ^]^ During the charge process, the Na_2_S_2_/Na_2_S was initially transformed to long‐chain NaPSs (Na_2_S_x_ (x = 4–8)), and then to sulfur, which was consistent with CV analysis above. The spectra collected during charging revealed opposite features to discharge, suggesting high reversibility of the polysulfide conversion on S@Se‐ZnS/HSC electrodes.

The electrochemical reaction mechanisms of the S@Se‐ZnS/HSC cathode were adequately revealed by the in situ XRD techniques, as illustrated in Figure 4g and Figure S24 (Supporting Information). A typical Na/S cathode would reveal the transition of crystalline α‐S_8_ (PDF no: 008–0247) to Na_2_S during the discharge process and finally into the β‐S_8_ phase (PDF no: 071–0137) during the charging process. However, in the S@Se‐ZnS/HSC cathode, due to the penetration of sulfur into the interior cavity and micro/mesopores, no distinct sulfur characteristic peaks were observed. Upon discharging from 1.5 V, characteristic peaks for the long‐chain Na_2_S_x_ (PDF no: 04‐003‐2049) have been observed, contributing only limited capacity. From 1.5 to 1.25 V, two new peaks were emerged at 22.37° and 35.66°, corresponding to Na_2_S_3_ (PDF no: 44–0822) and Na_2_S_4_(PDF no: 04‐007‐0591), respectively. As the discharge voltage decreased from 1.25 to 1.0 V, one peak at 39.3° appeared in XRD pattern. In addition, another broad peak ≈23° was assigned to the (111) plane of Na_2_S (PDF no. 47‐1689) and the (111) plane of Na_2_Se (PDF no: 47–1699). When fully discharged to 0.5 V, only a broad diffraction peak ≈23° corresponded to the final product of Na_2_S and Na_2_Se. During the first charge process, Na_2_S and Na_2_Se and most of the NaPSs are converted to β‐S_8_, with a small amount of NaPS remaining. The working process observed through in situ XRD techniques aligned with ex situ Raman results, demonstrating the stable and reversible polysulfides conversion.

### 2.4 Density Function Theory (DFT) Calculations

**Figure 5:**
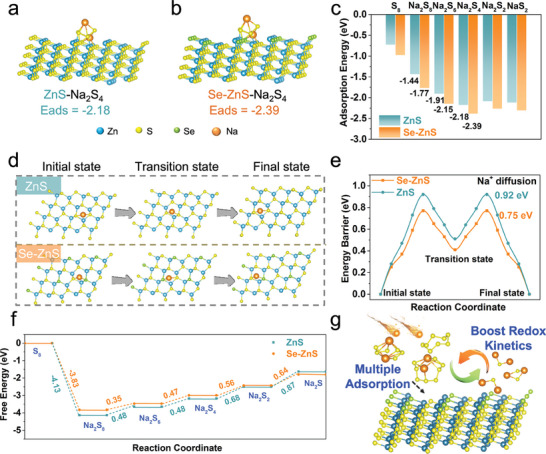
Density function theory (DFT) calculations: a) the optimized geometrical configurations of Na_2_S_4_‐ZnS. b) The optimized geometrical configurations of Na_2_S_4_‐Se‐ZnS. c) Adsorption energies for NaPSs on ZnS and Se‐ZnS surfaces. d) Total Na‐ion diffusion paths on the optimized (111) facet of ZnS and Se‐ZnS, respectively. e) Energy barrier of Na‐ion diffusion on (111) facet of ZnS and Se‐ZnS, respectively. f) Gibbs free energy profiles of NaPSs on ZnS and Se‐ZnS. g) Schematic illustration of the adsorbability and catalysis effect of Se‐ZnS toward NaPSs.

To get insight into the catalytic mechanisms and chemisorption capability of the S@Se‐ZnS/HSC cathode, density functional theory (DFT) calculations were employed. The optimal configuration of NaPSs binding on the Se‐ZnS (111) lattice plane are shown in **Figure** **5**a,b and Figures S25 and S26 (Supporting Information). The Se‐ZnS shows much higher adsorption energy for Na_2_S_4_, Na_2_S_6_, and Na_2_S_8_ (−2.39, −2.15, and −1.77 eV) than ZnS (−2.18, −1.91, and −1.44 eV, Figure 5c), which is consistent with the above UV absorption results. Furthermore, the diffusion barriers of Na^+^ on the Se‐ZnS (111) and ZnS (111) surface are simulated in Figure 5d,e. It can be found that Na^+^ on the Se‐ZnS (0.69 eV) has a smaller diffusion barrier than that on ZnS (0.77 eV), illustrating the facilitated Na^+^ transport brought by the modification of selenium in ZnS. Figure 5f reveals the calculated free energy of reduction from S_8_ to Na_2_S on the Se‐ZnS and ZnS in the discharging process.^[^ ^50^ ^]^ The solid–solid conversion from Na_2_S_2_ to Na_2_S shows the maximum energy barrier, indicating that it is the rate‐limiting step during the discharging process.^[^ ^51^ ^]^ The Gibbs free energy of Se‐ZnS (0.64 eV) in this step is lower than that of ZnS (0.87 eV), manifesting that the rate‐limiting conversion from Na_2_S_2_ to Na_2_S is thermodynamically more favorable on Se‐ZnS. Figure 5g schematically illustrates the catalytic effect of Se‐ZnS toward NaPSs, from which ZnS nanocrystals with modification of selenium atoms effectively facilitate the reduction from Na_2_S_2_ to Na_2_S, thereby significantly improving the electrochemical performance of sulfur cathodes and the utilization of sulfur.

### 2.5 Electrochemical Performance of S@Se‐ZnS/HSC Cathode at Subzero Temperature

**Figure 6:**
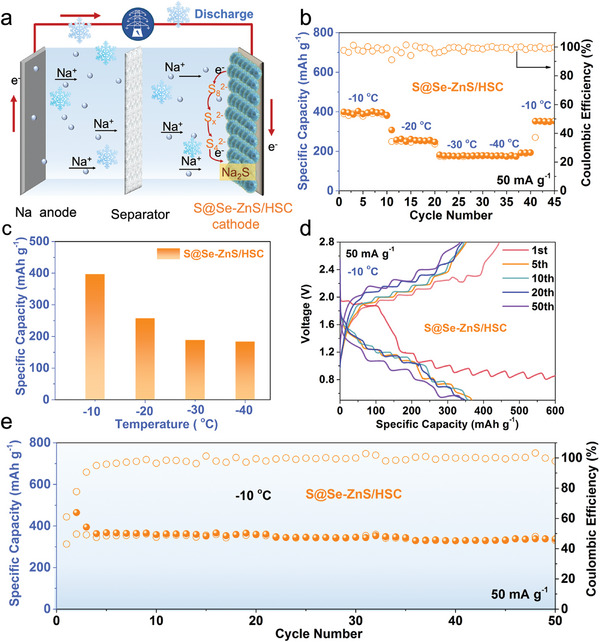
Electrochemical performance of Na–S battery at subzero temperature: a) schematic illustration of subzero temperature Na–S battery. b,c) Electrochemical performance of S@Se‐ZnS/HSC cathode at different subzero temperatures. d)The discharge/charge curves of S@Se‐ZnS/HSC cathode at 0.05 A g^−1^. e) The cycling property at 0.05 A g^−1^.

Due to the slow reaction kinetics of sodium‐ion energy storage systems, most sodium‐ion batteries do not work at low temperatures. This situation became even more serious since the reaction process of the Na/S battery undergoing various phase transitions. After tuning the electronic structure by the double modification of selenium, both the electrical conductivity and catalytic activity of the S@Se‐ZnS/HSC electrodes have been enhanced. Benefiting from this unique structure, the optimal electrodes exhibit the fastest conversion kinetics of polysulfides at room temperature, which is expected to achieve good electrochemical performance at low temperatures. Therefore, the cells with S@Se‐ZnS/HSC cathodes were tested under low temperatures. As shown in **Figure** **6**a, when the current density is 0.05 A g^−1^, the significant reversible capacity of 401.7 mAh g^−1^ for S@Se‐ZnS/HSC can still be reached at −10 °C (Figure 6b). When the temperature drops to −20 °C, the capacity of this cathode delivers a high specific capacity of ≈260.1 mAh g^−1^. As the temperature drops to −30 °C and −40 °C, the S@Se‐ZnS/HSC shows a specific capacity of 176 and 174 mAh g^−1^ (Figure 6c), respectively. As displayed in Figure 6d,e, the cycling performances of S@Se‐ZnS/HSC cathodes at −10 °C show a discharge capacity of 378.5 mAh g^−1^ over 50 cycles at 0.05 A g^−1^, with excellent cyclic capacity decays of 0.03%. The low‐temperature cycling performance of the S@Se‐ZnS/HSC cathode is considerably superior to those in the reported sub‐zero Na/S batteries.^[^ ^52^ ^]^ This phenomenon further demonstrates that the dual modulation of the electronic structure of sulfur and ZnS catalysts can effectively accelerate the conversion kinetics of polysulfide even at low temperatures.

## 3 Conclusion

In summary, we have demonstrated the selenium‐modulated ZnS in hollow carbon spheres for high sulfur utilization and fast‐kinetics RT Na–S batteries. The uniformly dispersed Se‐ZnS nanocrystals show a low reducing energy barrier and enhanced adsorption capacity and the hierarchical porous carbon exhibits well‐integrated conductivity and effective confinement for sulfur. Accordingly, the S@Se‐ZnS/HSC delivers good long‐cycle life (729 mAh g^−1^ after 100 cycles at 0.2 A g^−1^) and superior rate performance of 670.6 mAh g^−1^ at 5.0 A g^−1^. Besides, these S@Se‐ZnS/HSC cathodes show a discharge capacity of 378.5 mAh g^−1^ after 50 cycles at 0.05 A g^−1^ even at −10 °C. This dual‐regulation strategy of electric structure may shed light on the design of sulfur cathodes with enhanced conversion kinetics in practical Na/S batteries.

## 4 Experimental Section

### Synthesis of the Hierarchical Superstructure of MOF Nanorods

The MOF nanorods with about 7–9 µm were obtained by a self‐assembly method as follows: 40 mL of methanol solution containing 120 mg (0.6 mmol) of 2,5‐dihydroxyterephthalic acid was added into methanol solution (100 mL) of zinc acetate dihydrate (0.44 g, 2 mmol). After being stirred for 20 min, 20 mL of methanol solution containing 60 mg (0.3 mmol) of 2,5‐dihydroxyterephthalic acid was injected into the above solution. After sonicated for ≈30 min, the obtained precipitate was centrifugated three times. This prepared precipitates and 50 mg (0.84 mmol) of urea were dispersed in deionized water (30 mL). After ultrasonic treatment for 1 h, the green solution was hydrothermally treated at 175 °C for 24 h to form a spherical structure of MOF nanorods. The black‐yellow precipitate was obtained by washing with methanol and deionized water.

### Synthesis of HSC, ZnS/HSC, and ZnSe/HSC

The 300 mg of spherical structure MOF nanorods and 40 mg of selenium were placed on both sides of the ceramic boat and heated to 650 °C with a heating ramp rate of 3 °C min^−1^ in a tube furnace under an Ar+H_2_ (5%) flow for 1 h. After high‐temperature carbonization and selenization, ZnSe‐ZnO/HSC was obtained. The black powder was poured into 1.0 m HCl solution to remove ZnO. The precipitate was washed with deionized water and dried to get ZnSe/HSC samples. ZnS/HSC materials were prepared by vulcanization of ZnSe/HSC materials. 300 mg of ZnSe/HSC material and 300 mg of sulfur were transferred into a ceramic boat heated to 650 °C under an Ar flow for 1 h for vulcanization. To remove excess sulfur and redundant selenium, CS_2_ and ethanol solution (1:3, volume ratio) were used to wash the obtained powder. HSCs were obtained by high‐temperature carbonization of spherical superstructure MOF material but without the selenization process.

### Synthesis of S@HSC, S@ZnS/HSC, and S@Se‐ZnS/HSC

The vapor‐diffusion method was applied to load sulfur into the relevant porous composites. The ZnSe/HSC and sulfur powder were mixed at a weight ratio of 1:3 by ground milling and then transferred to a vacuum quartz tube. After that, this quartz tube was heated to 600 °C and then maintained for 6 h. After cooled to room temperature, the collected black material was annealed at 230 °C for 10 min under continuous N_2_ gas blowing to redundant sulfur removal outside of S@Se‐ZnS/HSC. For comparison, S@HSC and S@ZnS/HSC composites were prepared with similar procedures.

### Physical Characterizations

The morphologies of samples were measured by scanning electron microscope (at 3 kV, Ultra 55, Zeiss, Germany) and transmission electron microscope (at 200 kV, Tecnai G2 20 Twin, Fei, USA). The XRD patterns were taken using a Cu Kα radiation X‐ray diffractometer (XRD, X'pert PRO) at 20 mA and 40 kV. The Raman spectra were measured by Senterra R200‐L (Germany, λ = 532 nm). The sulfur content was conducted on a thermogravimetric analyzer (Pyris 1 TGA). XPS data were collected by a spectrometer (PHI 5300C). The pore size distribution and specific surface area of prepared materials were measured by an N_2_ adsorption device (Tristar II 3020, Micromeritics Instrument Corp.) was analyzed by Brunauer‐Emmett‐Teller (BET) method.

### Electrochemical Characterization

The S@HSC, S@ZnS/HSC, and S@Se‐ZnS/HSC samples were assembled into 2032‐type coin cells for electrochemical analyses. The electrode slurry was synthesized by mixing active material (70 wt.%), sodium carboxymethyl cellulose (CMC) (10 wt.%), and carbon black (20 wt.%). The above slurry was coated on an Al foil uniformly. The active material and sulfur loading on the aluminum foil was ≈2 mg cm^−2^ and ≈1 mg cm^−2^, respectively. Before being transferred to the glovebox, the electrodes were dried at 50 °C in a vacuum oven. The specific capacities of active material and current density were calculated on the basis of actual sulfur loading (TGA). The counter and reference electrodes in these coin cells were Na metallic. Here, 1.0 m NaClO_4_/EC/PC+FEC (volume ratio EC: PC = 1:1, 5.0 v% FEC) was used as the electrolyte with a glass microfiber membrane (Whatman, UK) as a separator. The cycling performance of active material in the potential range of 0.5–2.8 V at different current densities was tested on the LAND CT2001A test system (WuhanJinnuo Electronics, Ltd., China). The CV of active materials was conducted on the electrochemical workstations of CHI660E (the potential range: 0.5–2.8 V, the scan rate: 0.1 A g^−1^). Gamry Reference 6000 (Gamry Co., USA) electrochemical system was used to acquire cells’ impedance spectra, and the test frequency range was 100 kHz to 100 MHz.

## Conflict of Interest

The authors declare no conflict of interest.

## Supporting information

Supporting Information

## Data Availability

The data that support the findings of this study are available from the corresponding author upon reasonable request.
