# Supplementary material for: Enhancing Conversion Kinetics through Electron Density Dual‐Regulation of Catalysts and Sulfur toward Room‐/Subzero‐Temperature Na–S Batteries
Source: Adv Sci (Weinh). 2024 Apr 9;11(21):2308180. doi: 10.1002/advs.202308180 (PMC11151073; doi:10.1002/advs.202308180)
Supplement: Supplementary file 1 — Supporting Information [file ADVS-11-2308180-s001.pdf]

ADVANCED SCIENCE
Open Access

Supporting Information

for *Adv. Sci.*, DOI 10.1002/advs.202308180

Enhancing Conversion Kinetics through Electron Density Dual-Regulation of Catalysts and Sulfur toward Room-/Subzero-Temperature Na–S Batteries

Sainan Luo, Jiafeng Ruan, Yan Wang, Min Chen and Limin Wu**

Supporting Information

Enhancing Conversion Kinetics through Electron Density Dual-Regulation of Catalysts and Sulfur towards Room-/Subzero-Temperature Na-S Batteries

Sainan Luo, Jiafeng Ruan, Yan Wang, Min Chen, Limin Wu**

S. Luo, J. Ruan, Y. Wang, Prof. M. Chen, Prof. L. Wu
Department of Materials Science, Fudan University, Shanghai 200433 (P. R. China)
S. Luo
School of Materials and Chemistry, University of Shanghai for Science and Technology, Shanghai 200093 (P. R. China)

*E-mail: chenmin@fudan.edu.cn, lmw@fudan.edu.cn

Details of Theoretical calculations

All theoretical calculations were conducted by using Density Functional Theory (DFT) based on Vienna Ab initio Simulation Package (VASP). Perdew-Burke-Ernzerhof (PBE) functional was adopted to describe the electron interaction energy of exchange-correlation. The plane-wave cutoff energy was 500eV. The energy and force convergence criteria are 10^{-5} eV and 0.01 eV per angstrom, respectively. A 3×3×1 Monkhorst-Pack sampled k-points in the irreducible Brillouin zone for structure optimization. The Se-ZnS model with ZnS (111) surface

was obtained simply by replacing 1/9 sulfur atoms with selenium atoms in the ZnS. The vacuum region of 15 Å was set in the z-direction to avoid interference between adjacent layers. For the calculations of adsorption, the following formula (1) was used:

$$E_{ads} = E_{slab+A} - E_{slab} - E_A$$

While E_{ads} was the energy of adsorption, E_{slab+A} was the total energy of the slab which was adsorbed by polysulfide, E_{slab} was the energy of slab models and E_A was the energy of polysulfide.

For the calculations of Gibbs free energy, the following formula (2) was used:

$$G_{Na_xS_y} = E_{slab+Na_xS_y} + E_0 + nRT - TS + (2 - x)E_{Na} + (8 - y)E_S$$

While G_{NaxSy} was the Gibbs free energy, $E_{slab+NaxSy}$ was the total energy of the slab, (E_0+nRT-TS) were the zero-point energy correction, E_{Na} was the singlepoint energy of sodium atom and E_s was the single- point energy of sulfur atom. NaxSy was the shorthand notation of polysulfide (x=0, 2 and y= 1, 2, 4, 6, 8).

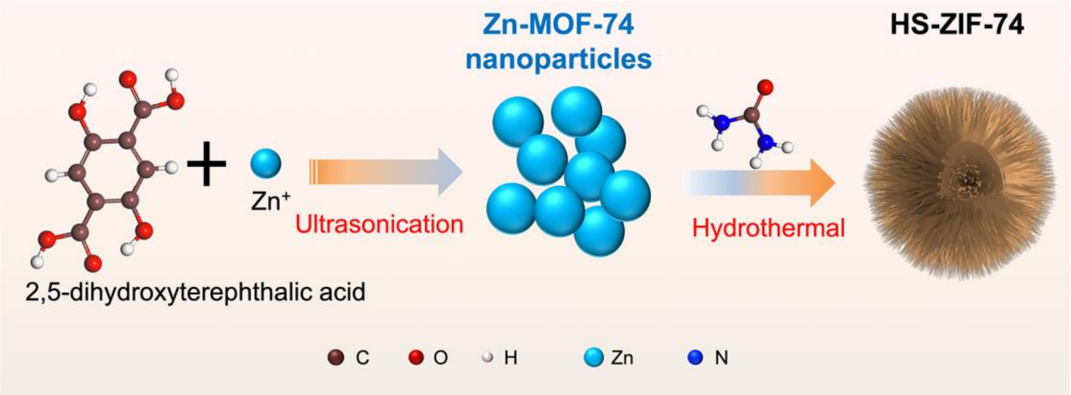

Figure S1. Schematic illustration of the formation of the HS-ZIF-74

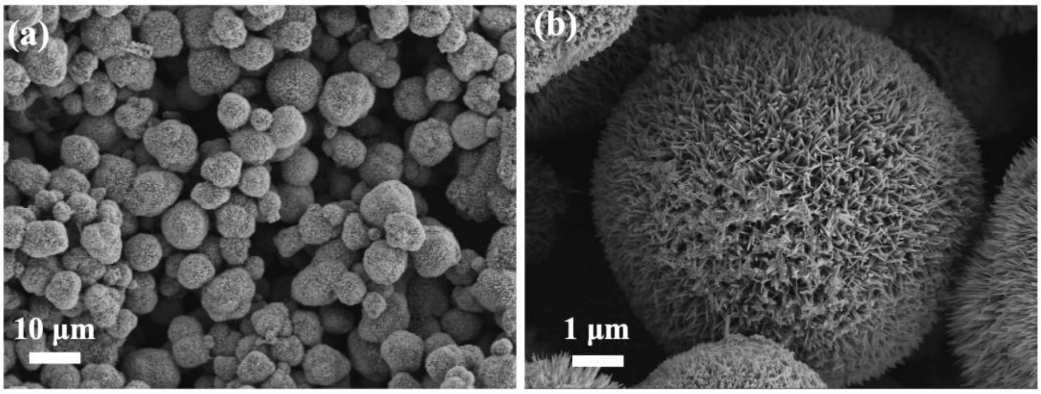

Figure S2. (a,b) FESEM images of HS-ZIF-74 .

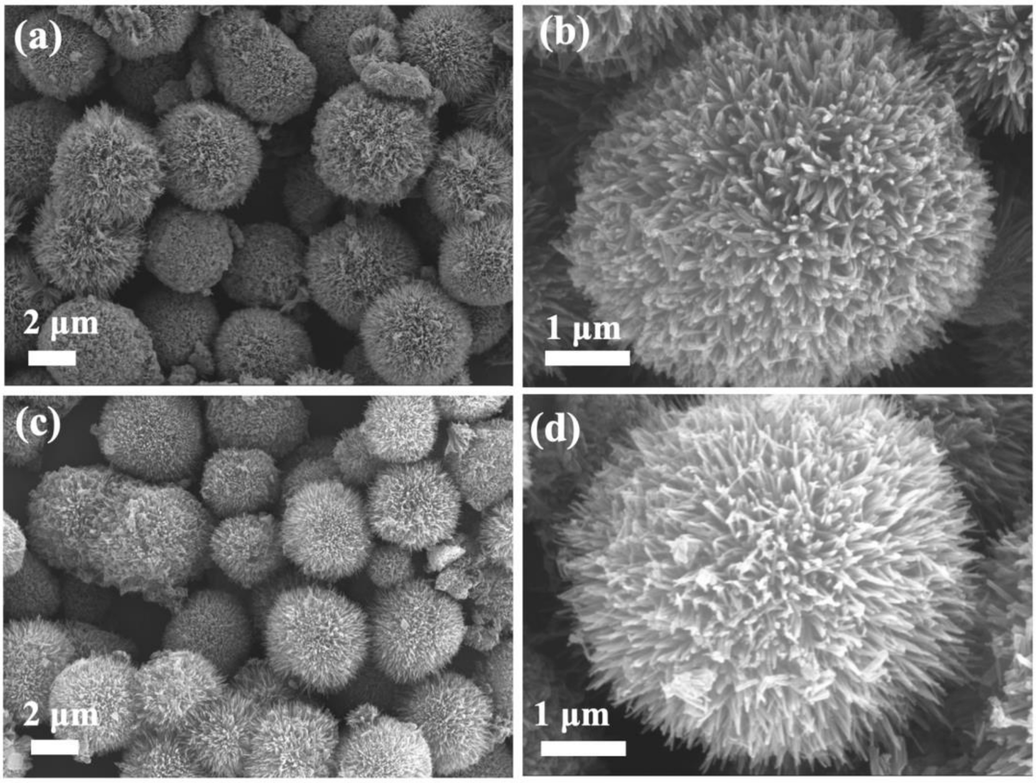

Figure S3. (a,b) FESEM images of S@HSC; (c,d) FESEM images of S@ZnS/HSC.

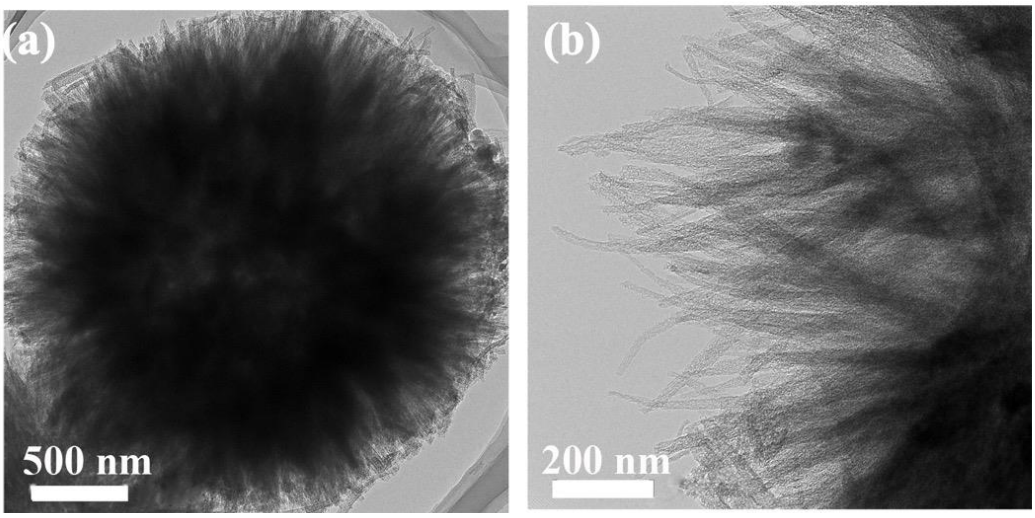

Figure S4. (a,b) TEM images of HSC.

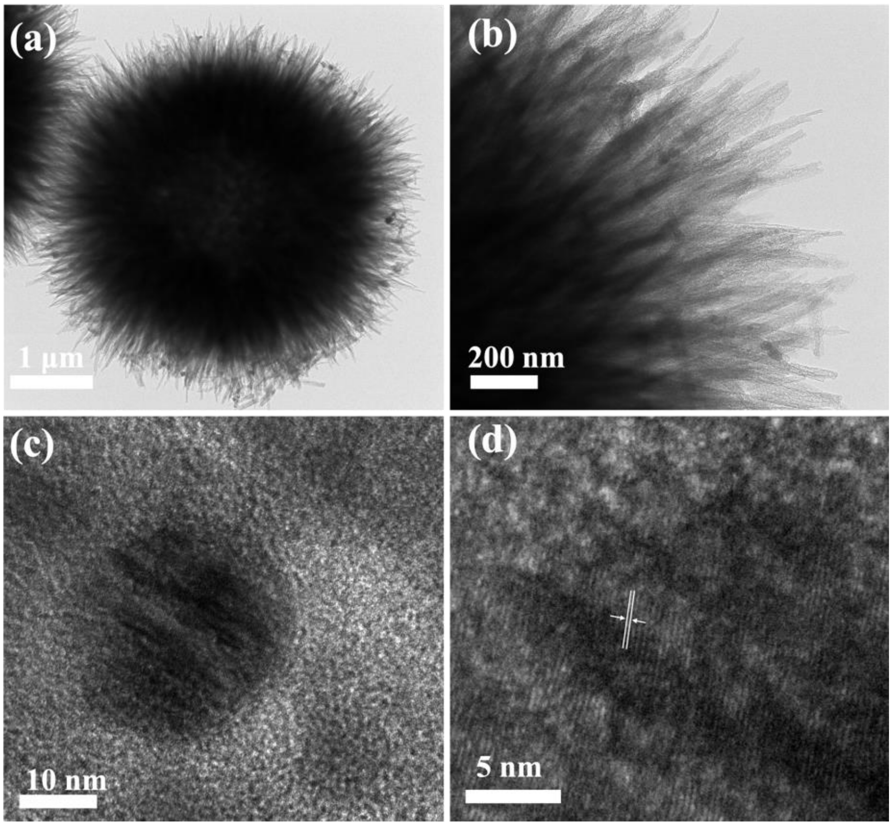

Figure S5. (a,b) TEM images of the ZnS/HSC material; (c,d) HRTEM images of the ZnS/HSC.

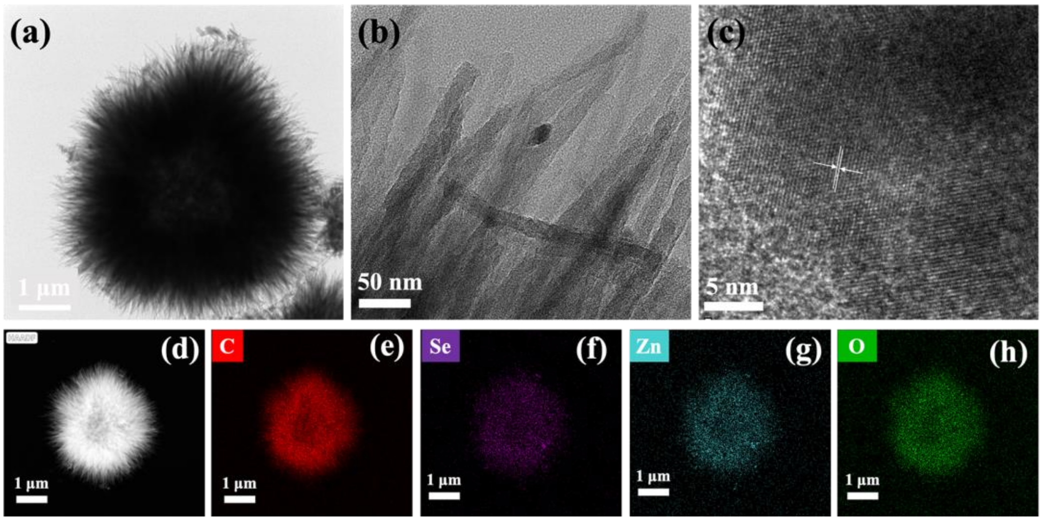

Figure S6. (a,b) TEM images of the ZnSe/HSC material; (c) HRTEM images of the ZnSe/HSC; (d-h)STEM micrographs and corresponding EELS chemical composition maps.

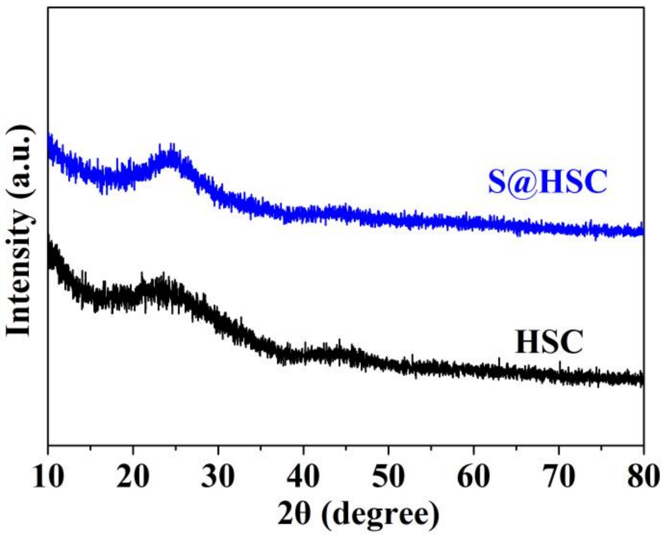

Figure S7. XRD patterns of HSC and S@HSC.

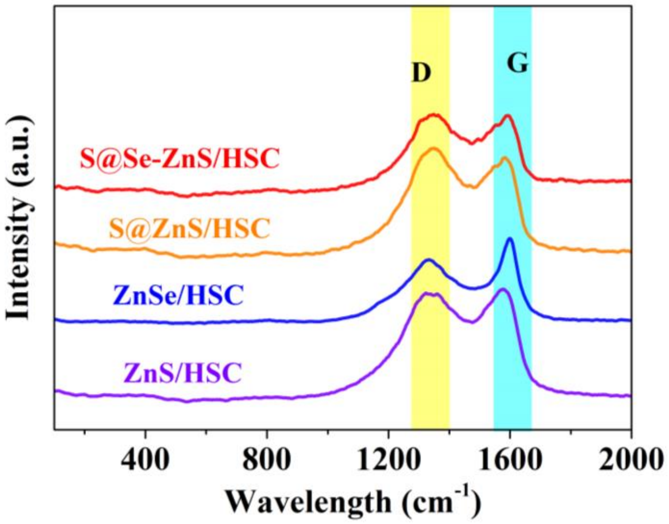

Figure S8. Raman spectra of ZnS/HSC, ZnSe/HSC, S@ZnS/HSC and S@Se-ZnS/HSC.

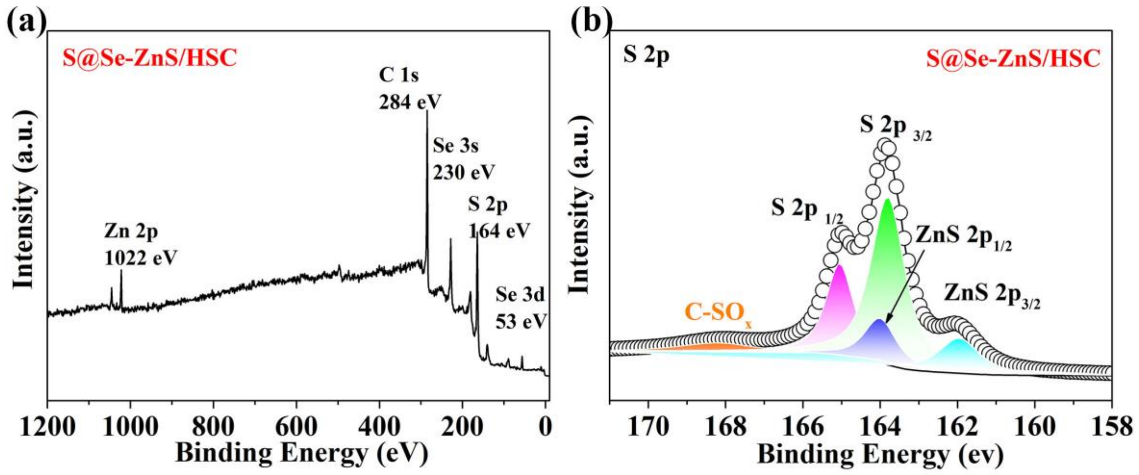

Figure S9. (a) The XPS survey spectrums of the S@Se-ZnS/HSC;(b) High-resolution XPS spectrum of S 2p in the S@Se-ZnS/HSC.

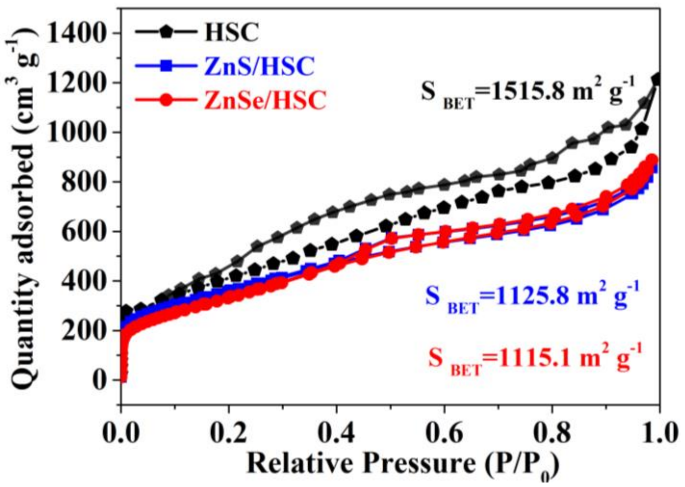

Figure S10. N_2 adsorption–desorption isotherms of the HSC, ZnS/HSC, and ZnSe/HSC.

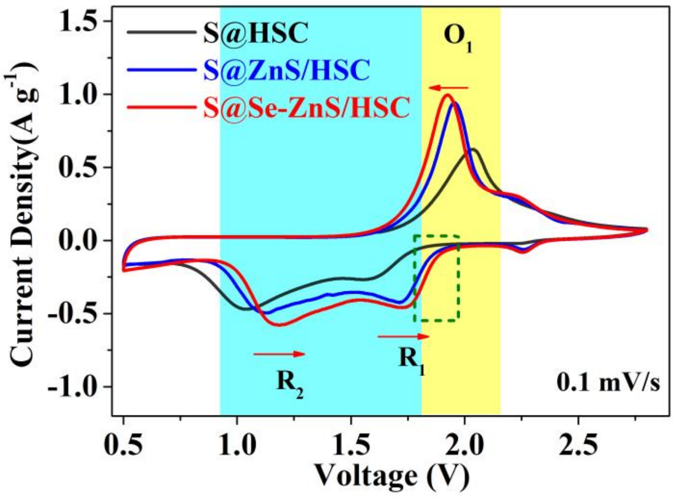

Figure S11. CV curves at a scan rate of 0.1 mV s^{-1}.

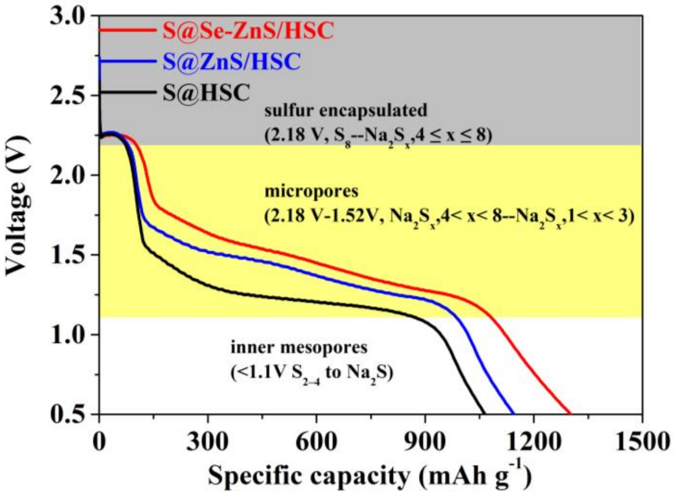

Figure S12. Galvanostatic discharge profiles of S@/HSC, S@ZnS/HSC, and S@Se-ZnS/HSC electrodes at first cycle at the current density of 0.1 A g^{-1}.

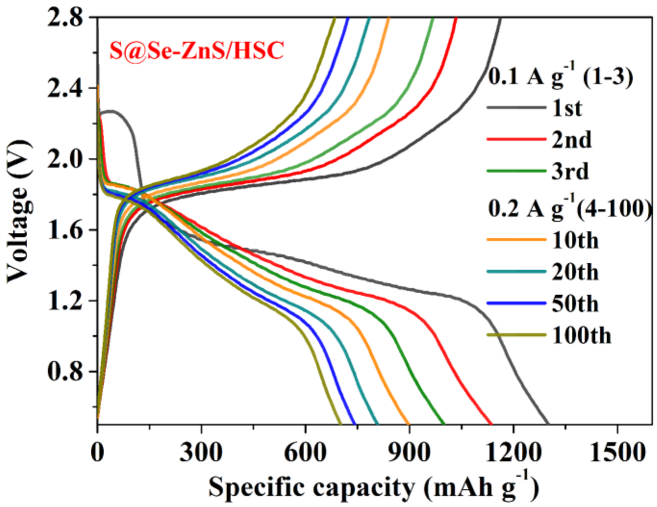

Figure S13. GDCcurves of S@Se-ZnS/HSC electrode at the current density of 0.2 A g^{-1}.

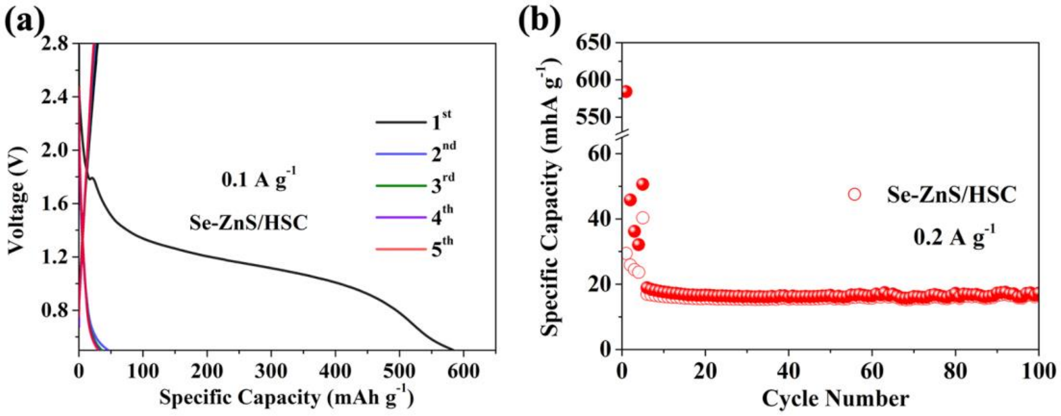

Figure S14. a) GCD curves of the Se-ZnS/HSC electrode at initial five cycles at the current density of 0.1 A g^{-1}. b) Cycling performance of the Se-ZnS/HSC electrode at the current density of 0.2 A g^{-1}.

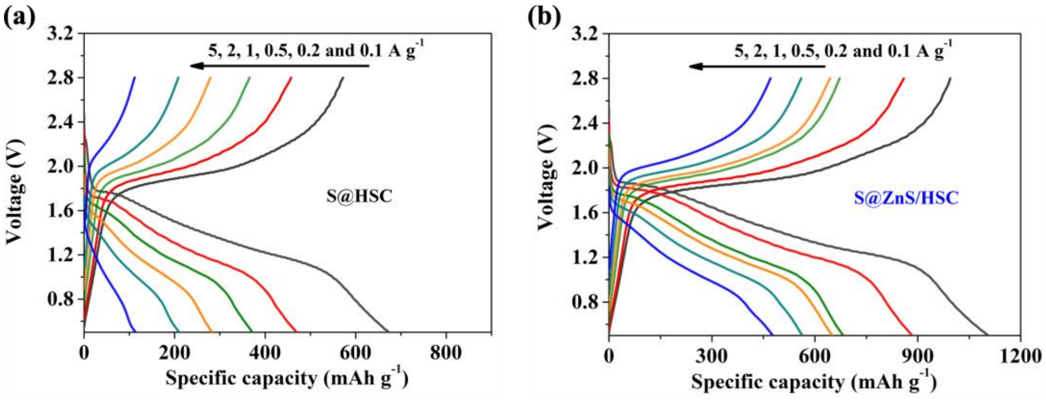

Figure S15. Charge/discharge curves S@/HSC, S@ZnS/HSC at different rates.

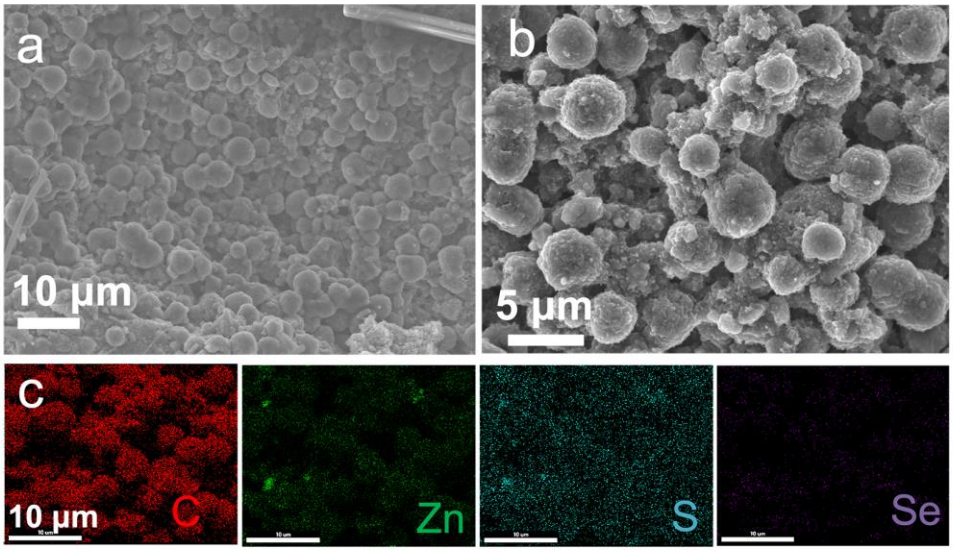

Figure S16. SEM images of a-b) S@Se-ZnS/HSC after long-term cycle. c) EDS elemental mapping of C, Zn, S and Se.

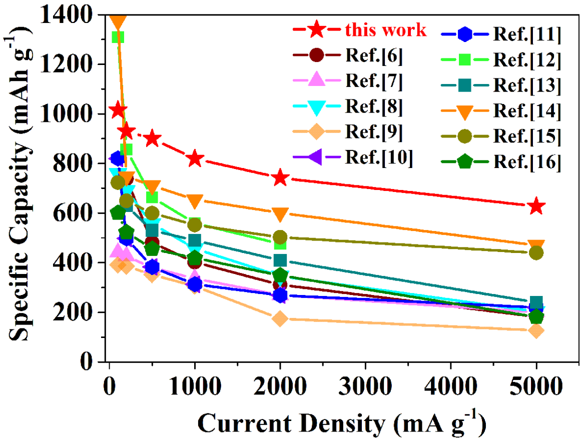

Figure S17. Rate comparison of this work and reported cathode for RT Na/S batteries

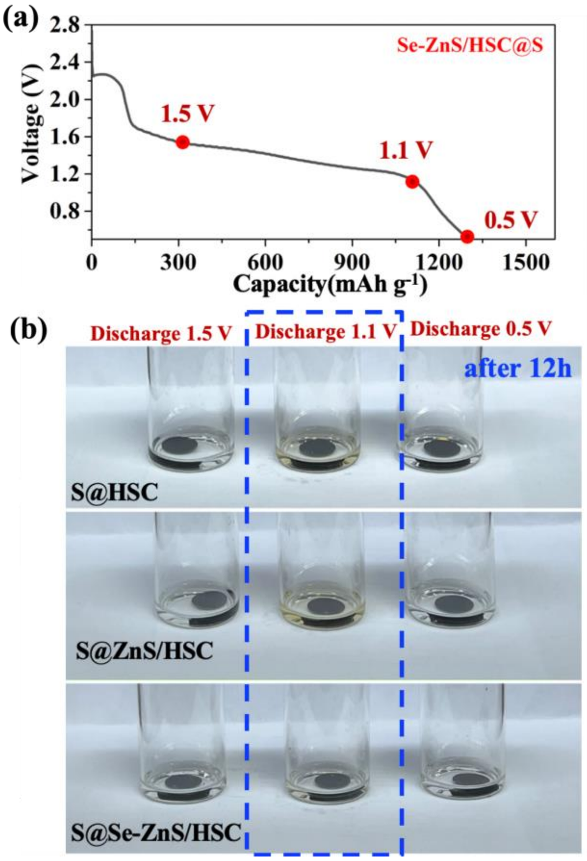

Figure S18. (a) Typical galvanostatic discharge profile marked with three different potentials, at which the coin cells of S@/HSC, S@ZnS/HSC, and S@Se-ZnS/HSC were disassembled, and their corresponding cathode electrode films were taken out and immersed in EC/PC solvent for 12 h; (b) Digital images.

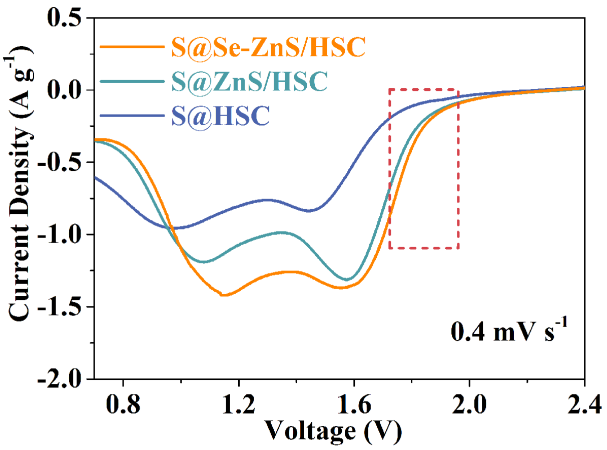

Figure S19 LSV curves at 0.4 mV s^{-1}.

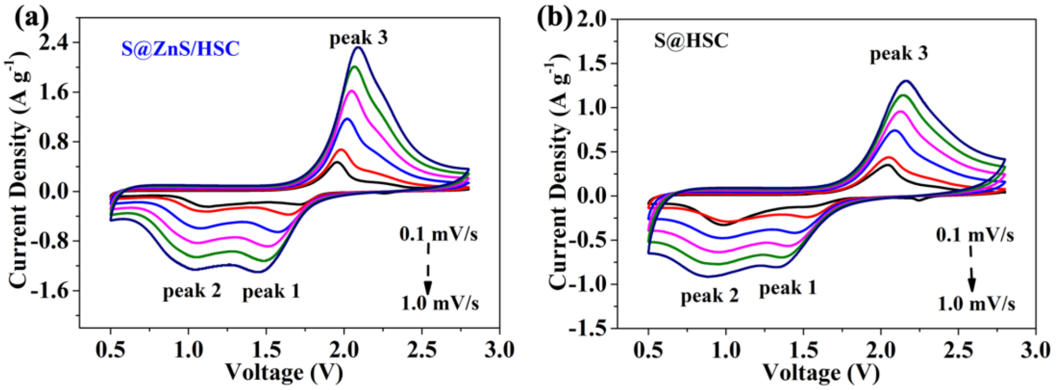

Figure S20. (a) CV curves of the S@/HSC cathode at 0.1, 0.2,0.4,0.6,0.8 and 1 mV s^{-1};(b) CV curves of the S@ZnS/HSC cathode at different scan rates;

The reaction mechanism can be determined by the calculation formulas of sweep rate (v) and peak (*i*) of CV :[12,13]

$$i = av^b \quad (1)$$

Where a and b are parameters to be determined (b = 0.5 and b = 1 represent d ion-diffusion-dominated behavior from faradaic intercalation and adsorption-dominated behavior from capacitive charge storage, respectively)[14].

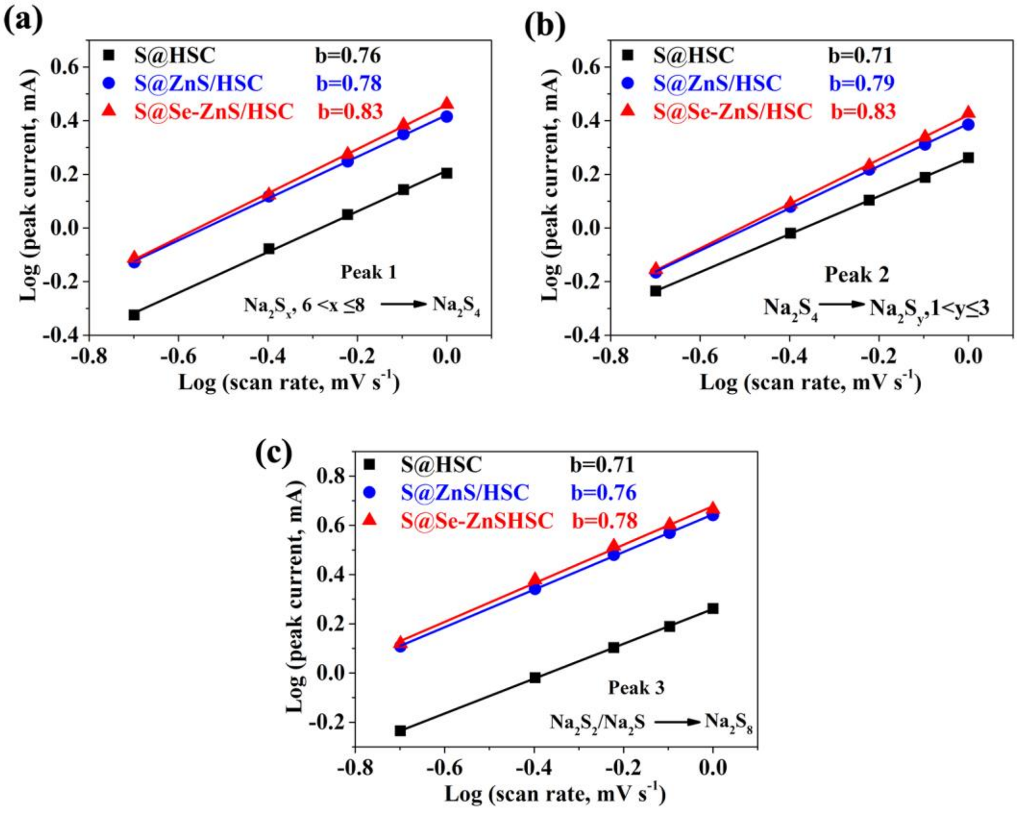

Figure S21. Line plots of log (peak current) versus log (scan rate) according to marked peak currents. (a) Line plots of log (peak1 current). (b) Line plots of log (peak2 current). (c) Line plots of log (peak3 current)

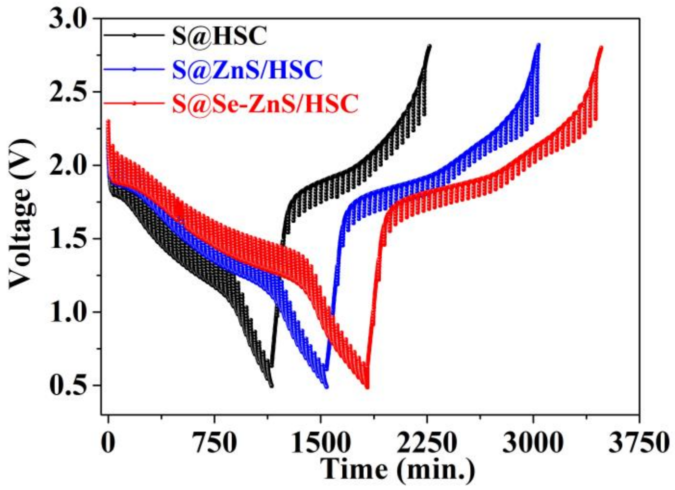

Figure S22. GITT curves at the first discharge/charge state of the S@/HSC, S@ZnS/HSC, and S@Se-ZnS/HSC electrodes.

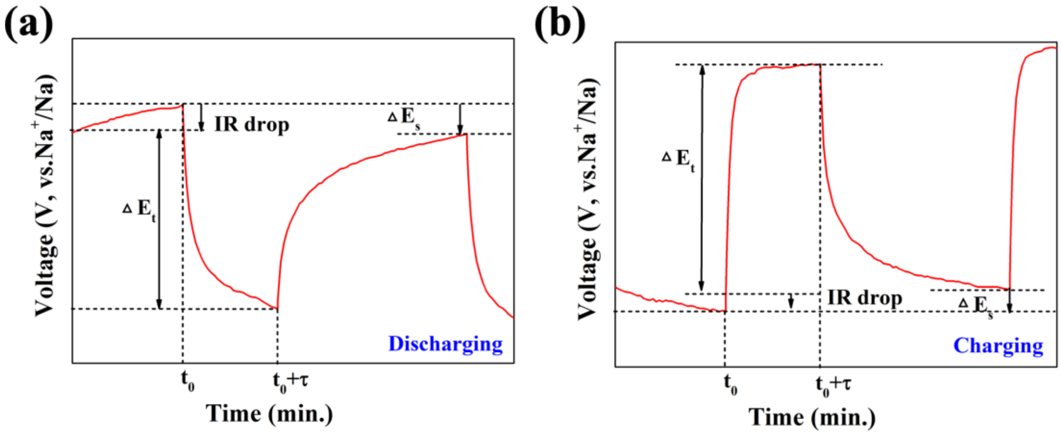

Figure S23. Schematic diagram of the important parameters in the GITT curve used to calculate the diffusion coefficient: (a) discharging and (b) charging.

$$D=\frac{4}{\tau\pi}\left(\frac{m_B V_M}{M_B S}\right)^2\left(\frac{\Delta E_s}{\Delta E_\tau}\right)^2 \qquad \left(t \ll \frac{L^2}{D}\right)$$

where τ is the current pulse time (s), mB and S are the active mass and geometric area for the tested electrode, m_B is the mass of the active material, M_B is the molar mass of the active material, V_B is the molar volume, and ΔE_S and ΔE_τ can be obtained from the GITT profiles[15,16].

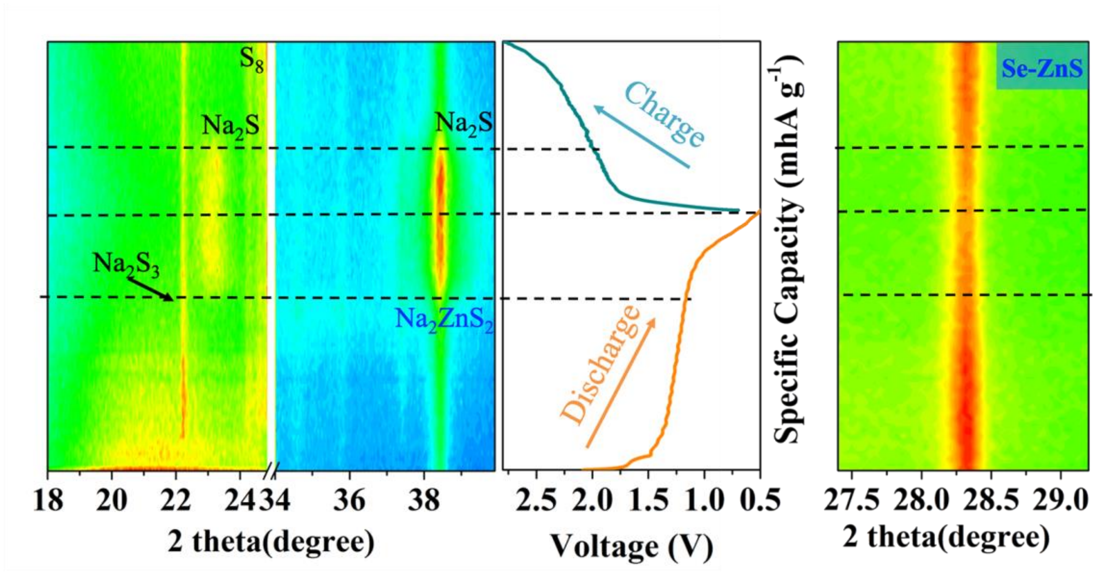

Figure S24. Reaction mechanisms of S@Se-ZnS/HSC: 2D contour plot of the in situ XRD within the first cycle

The Na_2S_x peaks did not vanish entirely from the diffractogram but remained discernible in the background. This suggested that a portion of the Na_2S_x adsorbed on the surface of glass fibers persisted permanently and did not participate in subsequent reduction and/or re-oxidation processes. From the contour plot in Figure S24, the peaks of Se-ZnS gradually diminished, replaced by emerging peaks at ~23.3° and 38.45° attributed to the formation of Na_2S and Na_2ZnS_2 (PDF no: 34-0946), respectively. Moreover, the similar working process is observed within the charge process, thus demonstrating the stable and reversible processes.

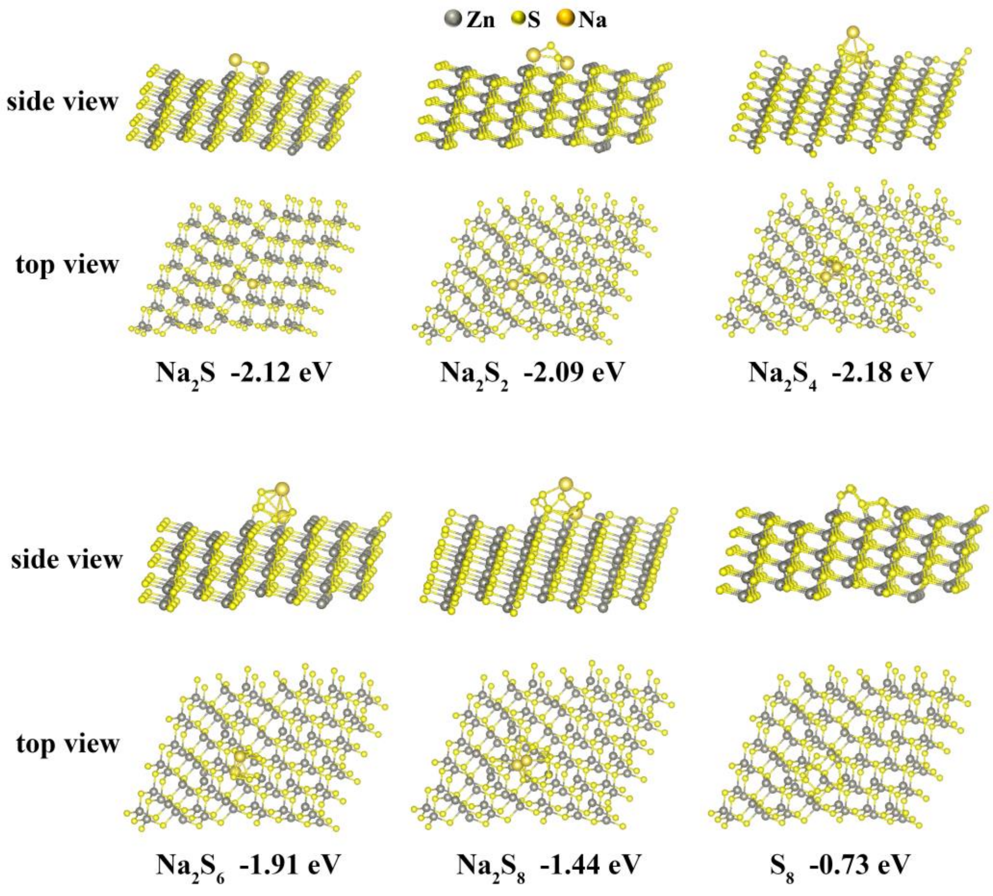

Figure S25. DFT calculation results of optimized geometrical configurations of the surface (111) of Se-ZnS with adsorbed NaPSs (Na_2S, Na_2S_2, Na_2S_4, Na_2S_6, Na_2S_8 and S_8).

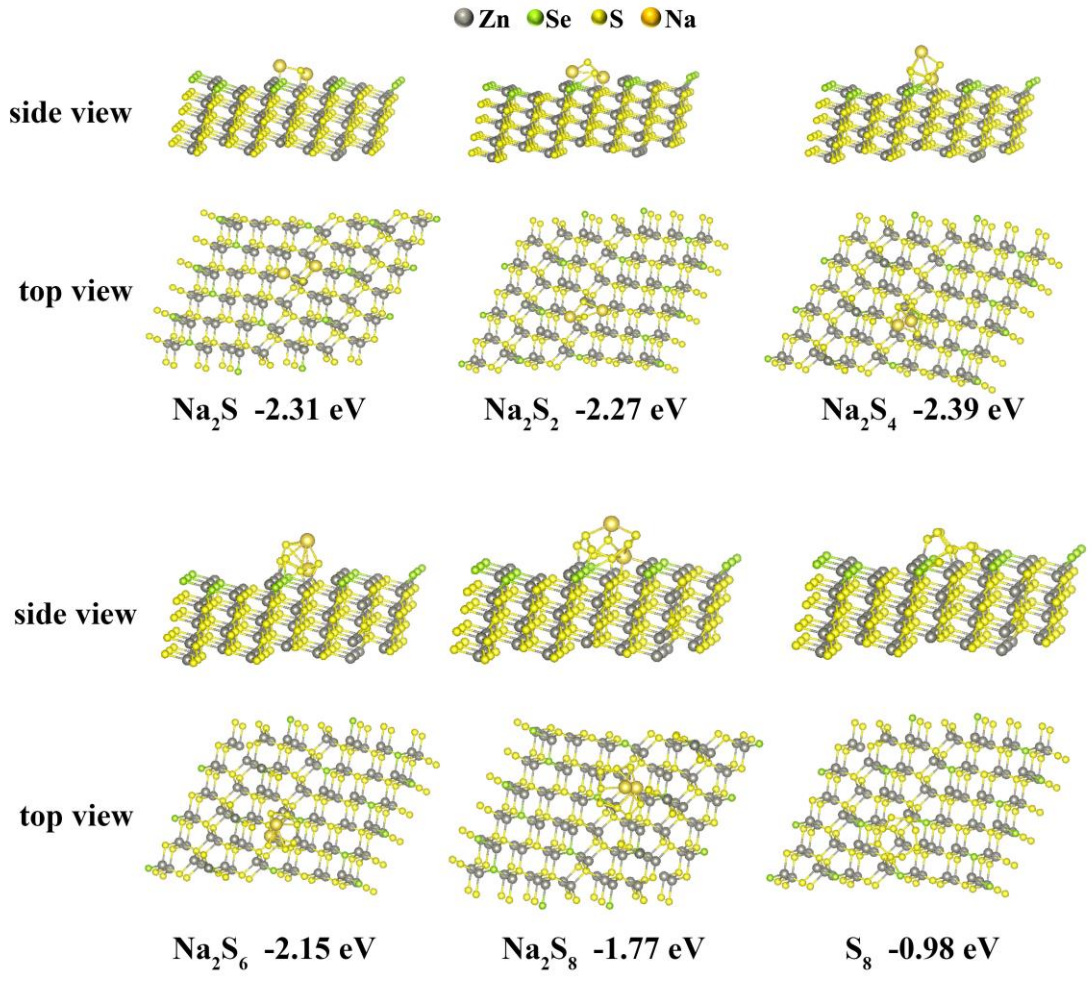

Figure S26. DFT calculation results of optimized geometrical configurations of the surface (111) of ZnS with adsorbed NaPSs (Na_2S, Na_2S_2, Na_2S_4, Na_2S_6, Na_2S_8 and S_8).

Table S1. Performance comparison of cathode materials for RT Na-S batteries

Modified material	Sulfur content (wt%)	Capacity (mAh g^{-1})/ current density	Capacity (mA h g^{-1}) /cycles	Reference
**S@Se-ZnS/HSC**	**68.2**	**869.2/1.0 A g^{-1}**		**This work**
		**670.6/5.0 A g^{-1}**	**477.5 /1000**	
S@Ni-NCFs	36	401.9 / 1.0 C	233 /270	1
		181.7 /5.0 C		

MOF-C/S/PDAc	37	336/1.0 A g^{-1}	270/1000	3
		201/5.0 A g^{-1}		
NiS_2@NPCTs/S	56	457/1.0 A g^{-1}	401/750	4
		203/5.0 A g^{-1}		
S@Fe-HC	40	313/1.0 A g^{-1}		6
		220/5.0 A g^{-1}		
S/MoS_2/NCS	43.8	654.3/1.0 A g^{-1}	360.7/2800	9
		470.7/5.0 A g^{-1}		
S/TiN-TiO_2@MCCFs	56.9	552.1/ 1.0 A g^{-1}	490.5 /500	10
		552.1/5.0 A g^{-1}	257.1/1000	
S@CoP-Co/NCNHC	53	419/1.0 A g^{-1}	448/700	11
		182/5.0 A g^{-1}		
FeS_2@NCMS/S	65.5	444 /1.0 A g^{-1}	395/850	17
		139/5.0 A g^{-1}		
S@$FeNi_3$@HC	50.1	867/1.0 A g^{-1}		18
		383/5.0 A g^{-1}		
S/MoN@CNFs	61	808/1.0 A g^{-1}		19
		500/5.0 A g^{-1}		
S@Co_1- CoS_2/NC	54	593/1.0 A g^{-1}		20
		433/5.0 A g^{-1}	357/5000	

Reference:

[1] B. Guo, W. Du, T. Yang, J. Deng, D. Liu, Y. Qi, J. Jiang, S. J. Bao, M. Xu, *Adv. Sci.* **2020**, 7, 1902617.

[2] S. Zheng, P. Han, Z. Han, P. Li, H. Zhang, J. Yang, *Adv. Energy Mater.* **2014**, 4, 1400226.

[3] F. Xiao, X. Yang, H. Wang, J. Xu, Y. Liu, D. Y. W. Yu, A. L. Rogach, *Adv. Energy Mater.* **2020**, 10, 2000931.

[4] Z. Yan, J. Xiao, W. Lai, L. Wang, F. Gebert, Y. Wang, Q. Gu, H. Liu, S. L. Chou, H. Liu, S. X. Dou, *Nat Commun* **2019**, 10, 4793.

[5] Y. X. Wang, J. Yang, W. Lai, S. L. Chou, Q. F. Gu, H. K. Liu, D. Zhao, S. X. Dou, *J. Am. Chem. Soc.* **2016**, 138, 16576.

[6] B. W. Zhang, T. Sheng, Y. X. Wang, S. Chou, K. Davey, S. X. Dou, S. Z. Qiao, *Angew. Chem., Int. Ed.* **2019**, 58, 1484.

[7] T. Yang, B. Guo, W. Du, M. K. Aslam, M. Tao, W. Zhong, Y. Chen, S. J. Bao, X. Zhang, M. Xu, *Adv. Sci.* **2019**, 6, 1901557.

[8] H. Yang, S. Zhou, B. W. Zhang, S. Q. Chu, H. Guo, Q. F. Gu, H. Liu, Y. Lei, K. Konstantinov, Y. X. Wang, S. L. Chou, H. K. Liu, S. X. Dou, *Adv. Funct. Mater.* **2021**, 31, 2102280.

[9] Y. Wang, Y. Lai, J. Chu, Z. Yan, Y. X. Wang, S. L. Chou, H. K. Liu, S. X. Dou, X. Ai, H. Yang, Y. Cao, *Adv. Mater.* **2021**, 33, 2100229.

[10] X. Ye, J. Ruan, Y. Pang, J. Yang, Y. Liu, Y. Huang, S. Zheng, *ACS Nano* **2021**, 15, 5639.

[11] Z. Yan, Y. Liang, W. Hua, X. G. Zhang, W. Lai, Z. Hu, W. Wang, J. Peng, S. Indris, Y. Wang, S. L. Chou, H. Liu, S. X. Dou, *ACS Nano* **2020**, 14, 10284.

[12] J. Ding, H. Zhang, H. Zhou, J. Feng, X. Zheng, C. Zhong, E. Paek, W. Hu, D. Mitlin, *Adv. Mater.* **2019**, 31, 1900429.

[13] X. Yang, J. Wang, S. Wang, H. Wang, O. Tomanec, C. Zhi, R. Zboril, D. Y. W. Yu, A. Rogach, *ACS Nano* **2018**, 12, 7397.

[14] B. Lin, X. Zhu, L. Fang, X. Liu, S. Li, T. Zhai, L. Xue, Q. Guo, J. Xu, H. Xia, *Adv. Mater.* **2019**, 31, 1900060.

[15] C. Lu, A. Li, G. Li, Y. Yan, M. Zhang, Q. Yang, W. Zhou, L. Guo, *Adv. Mater.* **2021**, 33, 2008414.

[16] J. Ruan, F. Mo, Z. Chen, M. Liu, S. Zheng, R. Wu, F. Fang, Y. Song, D. Sun, *Adv. Energy Mater.* **2020**, 10, 1904045.

[17] Z. Yan, Y. Liang, J. Xiao, W. Lai, W. Wang, Q. Xia, Y. Wang, Q. Gu, H. Lu, S. L. Chou, Y. Liu, H. Liu, S. X. Dou, *Adv. Mater.* **2020**, 32, 1906700.

[18] L. Wang, H. Wang, S. Zhang, N. Ren, Y. Wu, L. Wu, X. Zhou, Y. Yao, X. Wu, Y. Yu, *ACS Nano* **2021**, 15, 15218.

[19] Z. Li, C. Wang, F. Ling, L. Wang, R. Bai, Yu Shao, Q. Chen, H. Yuan, Y. Yu, Y. Tan, *Adv. Mater.* **2022**, 2204214.

[20] Y. Lei, C. Wu, X. Lu, W Hua, S Li, Y. Liang, H. Liu, W. Lai, Q. Gu, X. Cai, N. Wang, Y. Wang, S. Chou, H. Liu, G. Wang, S. Dou. *Angew. Chem. Int. Ed.* **2022**, 61, e202200384.
